# H:Q Ratios and Bilateral Leg Strength in College Field and Court Sports Players

**DOI:** 10.2478/v10078-012-0045-1

**Published:** 2012-07-04

**Authors:** Roy T.H. Cheung, Andrew W. Smith, Del P. Wong

**Affiliations:** 1 Department of Rehabilitation Sciences, The Hong Kong Polytechnic University, Hong Kong.; 2 Department of Health and Physical Education, The Hong Kong Institute of Education, Hong Kong.; 3 Department of Health and Physical Education, The Hong Kong Institute of Education, Hong Kong.

**Keywords:** asymmetry, soccer, volleyball, basketball, muscle imbalance, leg strength

## Abstract

One of the key components in sports injury prevention is the identification of imbalances in leg muscle strength. However, different leg muscle characteristics may occur in large playing area (field) sports and small playing area (court) sports, which should be considered in regular injury prevention assessment. This study examined the isokinetic hamstrings-to-quadriceps (H:Q) ratio and bilateral leg strength balance in 40 male college (age: 23.4 ± 2.5 yrs) team sport players (field sport = 23, soccer players; court sport = 17, volleyball and basketball players). Five repetitions of maximal knee concentric flexion and concentric extension were performed on an isokinetic dynamometer at two speeds (slow: 60°·s^−1^ and fast: 300°·s^−1^) with 3 minutes rest between tests. Both legs were measured in counterbalanced order with the dominant leg being determined as the leg used to kick a ball. The highest concentric peak torque values (Nm) of the hamstrings and quadriceps of each leg were analyzed after body mass normalization (Nm·kg^−1^). Court sport players showed significantly weaker dominant leg hamstrings muscles at both contraction speeds (P < 0.05). The H:Q ratio was significantly larger in field players in their dominant leg at 60°·s^−1^ (P < 0.001), and their non-dominant leg at 300°·s^−1^ (P < 0.001) respectively. Sport-specific leg muscle strength was evident in college players from field and court sports. These results suggest the need for different muscle strength training and rehabilitation protocols for college players according to the musculature requirements in their respective sports.

## Introduction

Muscle strength is one of the key factors in successful sports performance and is an important indicator of the effectiveness of injury rehabilitation in athletes. To monitor the performance of athletes as well as the rehabilitation progress of injured players, various lower limb strength indices have been investigated. Among these, hamstrings-to-quadriceps peak torque ratio (H:Q ratio) is one of the most commonly evaluated. This ratio of strength of agonist to antagonist knee muscles has been used to examine the functional ability, knee joint stability and muscle balance between hamstrings and quadriceps during velocity dependent movements (Aagaard et al., 1995; Clanton and Coupe, 1998; Hewett et al., 1996; 1999; Li et al., 1996; Orchard et al., 1997; Wong and Wong, 2009). Injury may occur during a rapid knee extension if the hamstrings fail to generate effective eccentric counteraction to decelerate the movement (Croisier et al., 2008). Also, when the hamstrings act to extend the hip, muscle strains may occur during rapid alternations between concentric and eccentric contractions (Petersen and Holmich, 2005). The anterior cruciate ligament (ACL), assisted by the hamstrings, stabilizes the knee by preventing anterior translation of the tibia on the femur (Kannus, 1988; Moore and Wade, 1989; Pettitt and Bryson, 2002), which can occur during pivoting movements such as landing from a jump and sudden changes in direction in field (soccer) and court (volleyball and basketball) athletes (Griffin et al., 2000). When the quadriceps generates substantially larger forces as compared to the hamstrings, excessive anterior translation may occur during dynamic activities, and the ACL will experience higher-than-normal shear forces. If the hamstrings are too weak to counteract this force, the ACL may be injured.

Typical concentric H:Q ratios in healthy subjects range from 0.5 to 0.8, with a higher ratios at faster angular knee velocities during isokinetic testing (Bennell et al., 1998; Clanton and Coupe, 1998; Grace et al., 1984; Orchard et al., 1997; Raunest et al., 1996). It has been found that athletes with a concentric H:Q ratio closer to 1.0 may have a reduced risk of hamstrings strain (Orchard et al., 1997). Also, a concentric H:Q ratio closer to 1.0 in athletes with ACL injury has been suggested to reduce the risk of an anteriolateral subluxation of the tibia (Li et al., 1996).

With respect to muscle strength in the dominant versus non-dominant leg, it has been suggested that there is an increased rate of injury when a difference of 15% or more in knee flexor or hip extensor strength occurs in collegiate athletes (Knapik et al., 1991). Likewise, greater discrepancy in bilateral leg muscle strength was found in two groups of injured softball players and track and field athletes (Newton et al., 2006; Yamamoto, 1993). Therefore, in addition to the issues of H:Q ratios within a subject’s leg, the discrepancy in peak torque production between dominant and non-dominant legs should also be investigated. It has been suggested that H:Q ratios and bilateral leg strength differences may indicate that leg muscle strength demands are sport-specific (Dvir, 2004a). College athletes who have high weekly training hours may present with asymmetry in muscle strength profiles due to specific technical skill requirements in particular sports (Anderson et al., 2003). For example, sports involving substantial jumping and running place a higher demand on the motor abilities of the hamstrings and quadriceps (Magalhaes et al., 2004). In addition, it has been shown that the injury rate of college athletes is comparable to professional athletes (Hoskins et al., 2003). However, there are no previous studies reporting H:Q ratios and bilateral strength differences between college athletes in field and court sports.

College athletes from field sports may present with a lower H:Q ratio as a result of higher sprinting demands in the sport. In contrast, athletes from court sports may require stronger hamstrings to compensate more frequent alteration between lower extremity acceleration and deceleration due to a relatively smaller playing area. The majority of previous investigations have focused on the evaluation of the indices in professional athletes in particular sports and subjects with ACL injury (Aagaard et al., 1997; Bennell et al., 1998; Gur et al., 1999; Harter et al., 1990; Kannus, 1988; Kramer et al., 1993; Read and Bellamy, 1990). However, studies examining healthy collegiate athletes and comparing the indices between field and court players are needed (Rosene et al., 2001). Therefore, our purpose was to compare H:Q ratios and bilateral differences in leg peak torque between healthy collegiate field and court players. Since the H:Q ratio is the most frequently-used variable for evaluating function in both athletes and patients with various injuries and pathologies of the knee, this study will provide both normative data and a testing model.

## Material and Methods

### Design

Our study adopted a cross-sectional design to investigate college athletes from field (soccer) and court (volleyball and basketball) sports. Subjects were recruited for our study by their fitness coach. During the competitive season (Oct 2009–Feb 2010), each of the athletes visited the Human Performance Laboratory where knee flexion and extension torque at two different angular velocities (60°·s^−1^ and 300°·s^−1^) on both legs were measured. The dominant leg was determined as the leg used to kick a ball (Spurrs et al., 2003). Subjects were instructed not to exercise vigorously 48 hours before the test to avoid any fatigue effects on the results.

### Subjects

A total of 40 male collegiate athletes (Table 1) were recruited from the intercollegiate soccer (n = 23), basketball (n = 5) and volleyball teams (n = 12), irrespective of their positional roles within the sport. All the participants were free from any active injury (no medical consultation or interruption of training in previous 6 months) and any previous surgery on the lower extremities. Our study was conducted according to the Declaration of Helsinki and the protocol was fully approved by the Human Research Ethics Committee before the commencement of the assessments. Written informed consent from all subjects was obtained following a brief but detailed explanation about the aims, benefits, and risks involved with this investigation. The subjects were told that they were free to withdraw from the study at any time without penalty.

### Isokinetic measurement

An isokinetic dynamometer (Cybex NORM, Cybex International, Inc., New York, USA) was used to measure knee flexion and extension torque at two different angular velocities (60°·s^−1^ and 300°·s^−1^). The dynamometer was calibrated prior to each test session according to the manufacturer’s standard machine protocol. We seated subjects on the dynamometer chair with chair back inclination angle of 85° (external angle from the rear horizontal) with stabilization straps at the trunk, abdomen and thigh to prevent excessive joint movements. The knee to be tested was aligned with the axis of the dynamometer, which was safeguarded by a fixed knob to prevent any possible hyperextension of the joint at fully extended position. Prior to data collection, the weight of the limb was normalized by in-built software for gravity correction.

In a pilot study, the test-retest reliability of this setup was calculated, which was excellent as indicated by the intraclass correlation coefficients of 0.99 (slow contraction) and 0.98 (fast contraction).

Each subject was given ample time to become familiarized with the experimental protocol on the isokinetic dynamometer before the test begun. Subjects underwent a standardized warm-up exercise following familiarization with the machine.

The warm-up exercise was composed of five submaximal and one maximal concentric contractions of the quadriceps and hamstrings muscles at the two test velocities. Following a three-minute rest, five repetitions of maximal knee concentric flexion and concentric extension were performed by the subjects at 60°·s^−1^ and 300°·s^−1^. The sequence of the leg being tested was counterbalanced and testing velocities were the same for all subjects. Between each test, three minutes of rest were given. The highest peak torque values of the flexors (hamstrings) and extensors (quadriceps) of each leg were analyzed after normalization by body mass. Bilateral muscle strength difference was defined according to a previous study as shown by the formula below (Yamamoto 1993).

Bilateral muscle strength difference = (normalized peak torque in dominant leg – non-dominant leg) / normalized peak torque of stronger leg

The testing velocities (60°·s^−1^ and 300°·s^−1^) were selected based on literature, which would allow for more meaningful comparisons with previously conducted studies. A slow testing velocity is usually regarded as one of the best indicators for the maximal peak torque output for a particular muscle group, whereas a high testing velocity simulates better the functional athletic movements (Dvir, 2004b). The H:Q ratio was calculated by dividing the concentric peak torque of hamstrings by that of quadriceps during the same contraction speed.

#### Statistical analysis

We used the normalized peak torques, bilateral leg strength differences and the H:Q ratios at each speed (60°·s^−1^ and 300°·s^−1^) for subsequent analyses. Differences in variables obtained from the isokinetic testing between groups (field sport versus court sport) were tested for significance by independent t-tests. There was no significant difference between the players from court sports, and therefore we grouped them together to represent court sports. Statistical significance was accepted at the P < 0.05 level. Data are presented as mean ± SD.

### Results

In our study, court players were significantly heavier than the field players (Table 1). Therefore, we normalized the peak torque values using body mass (Nm·kg^−1^) to eliminate the effects of subject heterogeneity in their body build. The normalized peak torque of hamstrings and quadriceps, and bilateral leg strength difference are presented in Table 2. No significant differences in normalized peak torque production between athletes of different sports were found in our data, except for court players who showed significantly lower dominant leg hamstrings strength in both slow (P = 0.011) and fast contraction (P = 0.015) speeds.

With regard to the discrepancy between dominant and non-dominant leg normalized peak torque production, our values ranged from 3% to 13% and there was no significant difference between field and court players (Table 2).

Our descriptive statistics for the H:Q ratios at different contraction speeds in field and court players are shown in Table 3. The H:Q ratio was significantly larger in the dominant leg of field players at 60°·s^−1^ (P < 0.001), and in their non-dominant leg at 300°·s^−1^ (P < 0.001) respectively.

## Discussion

The purpose of our study was to compare H:Q ratios and dominant versus non-dominant leg peak torque discrepancies in healthy collegiate field and court players. Isokinetic testing of the bilateral leg strength difference and H:Q ratio has been considered as a possible screening tool for injury risk (Bennell et al., 1998). Also during rehabilitation, these two indices are often used as a reference for treatment goal setting (Holm et al., 1994). It appears that healthy collegiate field and court players develop different muscular strength profiles according to our findings. We suggest that our results may indicate a need for a revision of the reference indices for injury risk assessment (Fousekis et al., 2011).

We believe it is logical to speculate that with long-term training effects, strength asymmetry may be present in some sports that involve unilateral movements. If bilateral leg strength difference is found to be different between healthy athletes of symmetrical and asymmetrical sports, adjustment of the 15% cut-off may be indicated

Furthermore, it has been found that athletes who perform predominantly unilateral movements are better in unilateral strength training exercises as compared to athletes who frequently perform bilateral movements (Wong et al., 2010).

Recently studies have proposed prediction formulae to increase the accuracy of load determination in lower-body strength training exercises and the hamstring muscle activation proportion during these exercises (Ebben, 2009; Ebben et al., 2008; Wong et al., 2010). Thus, in our opinion, attention to the exercise load and type should be paid when designing the exercise program for athletes from different sports.

In our study, both field and court players demonstrated less than 15% discrepancy between dominant and non-dominant leg muscle strength during isokinetic test and there was no significant difference between the two groups. The results of a study conducted by Rosene et al. (2001) that recruited 81 volleyball, soccer, basketball and softball players in similar skill level mirrored the results of our study. Rosene et al. (2001) found that side to side muscle strength difference was not demonstrated between athletes of the different sports. However, in another study involving 28 elite soccer and volleyball players (Magalhaes et al., 2004), the reverse trend was observed. In that study, bilateral hamstrings strength difference between dominant and non-dominant legs, during isokinetic test at 90°·s^−1^, was higher in soccer players. This pattern was explained by higher unilateral demands of hamstrings muscles in stabilizing actions in some specific soccer skills such as kicking and passing (Lees and Nolan, 1998). Similarly in our study, the field players did have significant stronger hamstrings strength in both contraction velocities than the court players. However, the bilateral leg strength difference was not statistically significant between field and court players.

Initially, we expected that soccer players would exhibit more asymmetrical leg differences due to the players predominantly kicking and passing the ball with their dominant leg (Lees and Nolan, 1998); and that volleyball players would be more symmetrical as their sport is dominated by such activities as blocking and spiking via vertical jumping with both legs (Gollhofer and Bruhn, 2003). We also expected that basketball players may demonstrate less bilateral leg strength imbalance than volleyball players due to the requirement of both single leg and double leg skills (Schiltz et al., 2009). In light of our findings, these assumptions on the symmetry of muscle demands in different sports may be oversimplified. Therefore, we suggest further study investigating the differences between elite and collegiate field and court athletes in muscle use during actual game situations. Also, we feel that there is a real need for a longitudinal study examining differences in injury risk amongst athletes of various sports over the course of entire seasons or even over the average length of the athlete’s career.

Published normal H:Q ratios range from 0.5 to 0.8 (Bennell et al., 1998; Grace et al., 1984; Raunest et al., 1996). The average H:Q ratios obtained from the recruited players in our study also fell into this range (0.53–0.82). Moreover, the H:Q ratios increased with higher testing velocity which agreed with previous studies (Hewett et al., 2008; Rosene et al., 2001). We found higher H:Q ratios (field sport = 0.63 ± 0.07; court sport = 0.53 ± 0.07; P < 0.0001) in the dominant leg of field players as compared to court players under slow contraction speed. We noted a similar pattern in the non-dominant leg under fast speed contraction (field sport = 0.82 ± 0.13; court sport = 0.64 ± 0.12; P < 0.0001). Similar results were reported in other recent studies showing higher H:Q ratios in soccer and rugby players than basketball players (Buchanan and Vardaxis, 2009; Metaxas et al., 2009).

The higher H:Q ratios shown in our field players can be explained by their stronger hamstrings. We suggest that the higher hamstrings peak torque production in the recruited field players (soccer players) may be related to the more frequent use of this muscle group to decelerate the lower leg during kicking and passing a ball (Lees et al., 2010). Initially, we expected higher quadriceps peak torque production in the court players because these sports (volleyball and basketball) may require more frequent vertical jumping. However, we did not observe this trend. The H:Q ratios we obtained from healthy field players were also found to be higher than previous studies that tested the subjects at the same angular velocities (Aagaard et al., 1995; Appen and Duncan, 1986; Richards, 1981; Wong and Wong, 2009).

From previous research, there is no consensus regarding H:Q ratios being sport-specific. Some previous studies reported no significant differences in H:Q ratio between different sports in elite and collegiate athletes respectively (Rosene et al., 2001; Zakas et al., 1995). However, Read and Bellamy (1990) found differences in the H:Q ratios between elite racket sports players and track athletes. Such conflicting findings were explained by the difference in training adaptations and the level of competition of the recruited athletes. However, we feel that further motion and muscle activation analyses of athletes in individual sports may be required for better explanation of conflict results in the previous reports.

As suggested by Aagaard et al. (1995), calculating a H:Q ratio using peak torques of eccentric hamstrings and concentric quadriceps contractions may be a better method in simulating the co-activation pattern developed in leg deceleration phase during the final range of motions of knee extension. However, in our study, the isokinetic testing protocol used the H:Q ratio between peak torques in concentric hamstrings and concentric quadriceps contractions. This allowed us to compare our data with a previous study of volleyball and soccer players (Magalhaes et al., 2004). To conduct isokinetic test with the eccentric hamstrings contraction, the subjects may encounter additional operational difficulties during tests, such as more complex coordination demands, longer measurement time and learning period required. In addition, eccentric testing has a higher potential for delayed muscle soreness and possible injury during the testing (Mair et al., 1995).

We have noted that there are some limitations of our study. Firstly, our investigation involved a single gender and generalization of data should be limited to males. Secondly, our H:Q ratios were obtained from concentric isokinetic test. Eccentric H:Q ratio may better reflect the injury mechanism between the agonist and antagonist. Finally, in our experiment, athletes were recruited from collegiate soccer, volleyball and basketball teams. We recommend that future studies recruit subjects from a wider variety of sports and from a range of levels from novice to elite/professional, which may better represent field and court sports.

## Conclusion

In our study, we presented sport specific requirements in muscle strength, represented by bilateral leg strength difference and H:Q ratio in healthy collegiate team field and court sport players. Based on our results, lower-body exercise programs designed for athletes from field versus court sports should consider (a) muscle activation ratio between quadriceps and hamstrings, and (b) the unilateral and bilateral nature of the training exercise that are suitable for that particular sport. Moreover, we recommend that, when setting rehabilitation goals or screening high-risk athletes at this competition level or higher, attention should be given to the specific sport played by the athletes.

## Figures and Tables

**Table 1:** Characteristics of subjects in the study

Sport	All	Field	Court
Age (years)	22.4 ± 1.6	22.2 ± 1.8	22.6 ± 1.4
Training density (hours/week)	6.7 ± 1.2	8	6
Experience in that sport (year)	7.2 ± 2.2	7.1 ± 2.2	7.4 ± 2.2
Body height (m)	1.75 ± 0.05	1.74 ± 0.05	1.75 ± 0.07
Body mass (kg)	66.89 ± 6.92	64.98 ± 5.07^*^	69.48 ± 8.30^*^
BMI (kg·m^−2^)	22.0 ± 2.01	21.4 ± 1.32	22.7 ± 2.55

* Significant difference between field and court players at P < 0.05.

**Table 2:** Muscle strength differences between field and court players

Sport	Muscle / Velocity	Dominant leg (Nm·kg^−1^)	Non-dominant leg (Nm·kg^−1^)	Difference (%)
Field	Hamstrings			
	60° ·s^−1^	1.89 ± 0.25 ^*^	1.68 ± 0.31	10.32 ± 15.29
	300° ·.s^−1^	1.04 ± 0.19 ^*^	0.91 ± 0.18	12.57 ± 12.96
	Quadriceps			
	60°·.s^−1^	3.03 ± 0.42	2.91 ± 0.38	3.41 ± 11.25
	300°·˙s^−1^	1.30 ± 0.25	1.23 ± 0.21	4.64 ± 15.04
Court	Hamstrings			
	60° ·s^−1^	1.68 ± 0.24 ^*^	1.61 ± 0.34	4.87 ± 12.78
	300°·˙s^−1^	0.88 ± 0.21 ^*^	0.83 ± 0.24	5.95 ± 25.86
	Quadriceps			
	60°·s^−1^	3.18 ± 0.54	2.87 ± 0.48	9.12 ± 14.16
	300°·s^−1^	1.37 ± 0.24	1.29 ± 0.26	5.42 ± 17.97

* Significant difference between field and court players at P < 0.05.

**Table 3:** The ratio between hamstrings and quadriceps strength (H:Q ratio)

Sport	Velocity	Dominant leg	Non-dominant leg
Field	60° ·s^−1^	0.63 ± 0.07 ^*^	0.58 ± 0.08
	300° ·s^−1^	0.75 ± 0.13	0.82 ± 0.13 ^*^
Court	60° ·s^−1^	0.53 ± 0.07 ^*^	0.57 ± 0.12
	300° ·s^−1^	0.65 ± 0.17	0.64 ± 0.12 ^*^

* Significant difference between field and court players at P < 0.05.
